# Arsenic Poisoning due to High Consumption of Canned Sardines in Jeddah, Saudi Arabia

**DOI:** 10.7759/cureus.12780

**Published:** 2021-01-19

**Authors:** Leen Othman, Abeer Nafadi, Saleh H Alkhalid, Nadia Mazraani

**Affiliations:** 1 Medicine, King Abdulaziz Medical City, King Saud Bin Abdulaziz University for Health Sciences, Ministry of National Guard, Jeddah, SAU; 2 Family and Community Medicine, King Abdulaziz Medical City, King Saud Bin Abdulaziz University for Health Sciences, Ministry of National Guard, Jeddah, SAU

**Keywords:** arsenic, toxicosis, poisoning, heavy metals, seafood, sardines, neuropathy

## Abstract

Dietary consumption of arsenic is considered the largest source of toxicosis for nonoccupationally exposed individuals as it can be ingested through contaminated underground water, seafood, animal products, and crops irrigated with polluted water. We present a case of a 45-year-old Caucasian male who had headaches and peripheral neuropathy for more than two months. He suspected arsenic poisoning as he has a regular heavy consumption of seafood and canned sardines. Analysis of urinary arsenic concentration confirmed his intoxication with arsenic. Yet, since it was of an inorganic form, he was prescribed with symptomatic treatments only. A symmetrical sensory or motor polyneuropathy featuring more distal impairment is among the most common neurological findings induced by arsenic toxicosis. Hence, a presenting history of heavy seafood diet should raise the differential diagnosis of arsenic or heavy metal poisoning and to investigate accordingly. This case illustrates the importance of taking the dietary regime of any patient presenting with neuropathy. In addition, the authorities must impose further rigorous surveillance and strict safety measures on food products and staples to minimize any sources of contamination of any sorts.

## Introduction

Arsenic is a natural chemical element of the earth’s crust that is highly distributed in the environment, especially underground water as it seeps into it. The inorganic form, or man-made formulas used in agriculture, mining, and manufacturing are particularly responsible for arsenic poisoning [1]. High levels of ingestion or inhalation of arsenic results in acute symptoms of arsenicosis. Initially, skin changes (warts and redness) occur, then other manifestations such as abnormal heart rhythm, upper and lower extremity tingling, nausea, vomiting, and diarrhoea occur [1]. Arsenic can be ingested through contaminated underground water, seafood, animal products, and crops irrigated with polluted water [2]. Though the United States, India, China, and Mexico are among the countries with highest levels of arsenic-containing groundwater, Saudi Arabia is not known for groundwater drinking [2]. Although arsenic poisoning is under-investigated in the Kingdom, we report the first case of arsenic poisoning in 2018 at Jeddah, Saudi Arabia. The study is approved by the Institutional Review Board (IRB) of King Abdullah International Medical Research Center (KAIMRC).

## Case presentation

A 45-year-old Caucasian male presented to the outpatient clinic with a chief complaint of chronic headaches for more than two years. He described it to be dull, intermittent (four to five times per month) and in the occipital region. In addition, sometimes it is pulsating at the frontal region and is associated with noise and light sensitivity. He reported no loss of consciousness, cognitive decline, nausea, or vomiting. However, two months after the first presentation he developed progressive bilateral numbness in his upper extremities and feet.

The physician conducted a thorough neurological examination and reported bilateral tremors in his hands. Otherwise there was no papilloedema, no relative afferent pupillary defect (RAPD), equal and reactive pupils, normal extra ocular muscles, normal cognition, tone, reflexes, and gait and no cerebellar or resting tremors. He is vitally stable but hypertensive (144/82 mmHg), has central obesity, and a BMI of 33.61 kg/m2. His past medical history includes hypertension, dyslipidemia, and obstructive sleep apnoea (OSA). His systemic review was unremarkable as there were no gastrointestinal symptoms, severe fatigability, weakness, sleep disturbance, hair or weight loss, rash, erythema, or fever. He is a healthcare worker with night on-calls three times a week. 

The patient was concerned of arsenic or lead poisoning given his diet which consisted of heavy consumption of canned sardines and seafood in general, yet he cannot determine the duration of exposure. His worry is further aggravated due to the fact that his family members were experiencing similar symptoms within the same time frame. 

Differential diagnoses involved mixed headaches due to his OSA, migraine, or heavy metal toxicosis. The lab work included full blood count (FBC), heavy metal screening, urine analysis of lead and arsenic, and magnesium levels. Additionally, he ordered brain MRI, brain CT, abdominal X-ray, nerve conduction study (NCS), and electrocardiogram (ECG). The results came back unremarkable as there were no signs of toxic encephalopathy in the MRI, and no radio-opaque densities in the abdomen that might indicate arsenic deposits. The NCS was normal for both upper and lower extremities and the ECG showed normal sinus rhythm. His laboratory work was of normal measures except for elevated urine arsenic levels. It was 22+ ug/L (normal range <15 ug/L) yet unspecified if organic or inorganic. Another urine arsenic analysis was requested with the instructions of not consuming seafood. The result for the second urine arsenic analysis came back as 20+ ug/L. 

Regarding the management protocol, the literature recommends treatment of only inorganic arsenic poisoning; so the patient was prescribed symptomatic treatments only and regular follow up. His list of medications included topiramate (50 mg twice a day), acetaminophen (500 mg oral prn), naproxen (500 mg oral prn), ibuprofen (400 mg oral prn for 12 days), and diclofenac 1% emulgel (50 g for 90 days).

## Discussion

The clinical manifestations of acute arsenic toxicosis include skin changes, abnormal heart rhythm, upper and lower extremity neuropathy, and gastrointestinal symptoms [1]. In addition, chronic symptoms due to long-term exposure advance to skin darkening, constant sore throat, and persistent digestive issues [1]. A characteristic white striae on the fingernails are suggestive of arsenic polyneuritis despite the fact that arsenic levels in hair and urine might be within normal ranges [3]. These symptoms may start presenting within five years of intermittent contact and induce some complications of arsenic-related malignancies such as bladder, blood, and skin cancer in addition to other health problems like diabetes, cardiac diseases, and neurotoxicity [1]. A symmetrical sensory or motor polyneuropathy featuring more distal impairment is among the most common neurological findings induced by arsenic toxicosis [4].

Dietary consumption is the largest source of exposure for nonoccupationally exposed individuals as it can be ingested through contaminated underground water, seafood, animal products, and crops irrigated with polluted water [5]. Though we already established that Saudi Arabia is not known for groundwater drinking, nevertheless, its source of arsenic poisoning can mainly be from contaminated rice as a study found that 37 brands of imported rice commonly consumed in Saudi Arabia have arsenic levels above the acceptable regulatory limits irrespective of soaking or rinsing [6]. Moreover, a study conducted in the Najran area of the Kingdom determined the concentrations of arsenic in four staple foods (rice, wheat, red meat, and chicken) and also found elevated levels of arsenic in them [7].

With regard to seafood, almost most fish and shellfish are heavily rich in arsenobetaine, which is the organoarsenic compound that is responsible for the total urinary arsenic level. Scallops, mussels, and some seaweed are also full in arsenosugars. They are metabolized to several compounds (mainly dimethylarsinic acid) that also add to the total urinary arsenic level [8]. The estimated arsenic concentration in seafood in general is averaged four to five part per million (ppm) [9], which is markedly higher than concentrations found in grains and cereals, with an average of 0.02 ppm [10].

Diagnosing arsenicosis happens by measuring elevated arsenic levels in the blood, fingernails, hair, or urine. Although a 24-hour urine test is mostly used with acute exposure cases (few days), according to the United States Centers for Disease Control and Prevention (CDC), the remaining investigations assess the long-term exposure of minimally six months [2]. For instance, NCS will show prominent electrophysiological findings such as pronounced abnormalities in both sensory and mixed nerve conduction and moderate irregularities in motor conduction [4]. Literature review described an acute or subacute demyelinating polyneuropathy starting one to three weeks following arsenic exposure [11]. Despite that, these tests only estimate the high amount of arsenic in the body and cannot determine any imminent adverse effects from exposure [2].

Our literature review found only one reported case of arsenic poisoning in 2015 in Riyadh, Saudi Arabia. It presented a 39-year-old woman who was on a gluten-free diet for eight years because of her celiac disease. She presented with loss of appetite, abnormal taste, diarrhoea, headache, insomnia, and impaired short-term memory and concentration although without skin lesions. Her 24-hour urine test of arsenic concentration was 682.77 µg/g and her creatinine level was within the normal range. She responded to chelation therapy with oral dimercaptosuccinic acid and recovered in two weeks. The suspected source of her arsenic poisoning was rice as she had not eaten seafood nor drank contaminated water [12].

Our patient, however, presented with mild neuropathy in the absence of other systemic manifestation of arsenic poisoning. It is speculated that the severity of the peripheral neuropathy and the absence of systemic symptoms are determined by the amount of toxic material ingested and the duration of exposure. Also, individual factors such as host variability in the hepatic P450 system may contribute to the acuteness of neuropathy and clinical presentation. The pathophysiology of arsenic poisoning is not well understood, but it has been suggested that arsenic is primarily destructive to the cell body as segmental demyelination occurs prior to axonal degeneration [11].

The present case was limited as we could not track down the canned sardines to test for the arsenic levels even though our testing and analysis of the inorganic arsenic concentration constitute evidence of exposure to arsenic. Additionally, the patient was not approachable to gain further clarifications regarding some symptoms and investigations.

## Conclusions

In summary, we presented a case of headache and neuropathy due to canned sardines that were consumed regularly by our patient and his family. The diagnosis of arsenic poisoning was confirmed by his elevated urinary arsenic concentration. Since his source was inorganic, the therapy regime consisted of symptomatic treatment in which it was successful and sufficient for our patient. He resumed his work in the healthcare field shortly after commencing the treatments without further complaints. This case illustrates the importance of taking the dietary regime of any patient presenting with neuropathy. Also, the authorities must impose further rigorous surveillance and strict safety measures on food products and staples to minimize any sources of contamination of any sorts. 
